# WNT5A from the fetal liver vascular niche supports human fetal liver hematopoiesis

**DOI:** 10.1186/s13287-021-02380-z

**Published:** 2021-06-05

**Authors:** Yoon Jung Choi, Adam M. Heck, Brian J. Hayes, Daniel Lih, Samuel G. Rayner, Brandon Hadland, Ying Zheng

**Affiliations:** 1 Department of Bioengineering, University of Washington, Seattle, WA USA; 2 Clinical Research Division, Fred Hutchinson Cancer Research Center, Seattle, WA USA; 3 Division of Pulmonary, Critical Care and Sleep Medicine, University of Washington, Seattle, WA USA; 4 Department of Pediatrics, University of Washington School of Medicine, Seattle, WA USA; 5 Center for Cardiovascular Biology, and Institute of Stem Cell and Regenerative Medicine, University of Washington, Seattle, WA USA

**Keywords:** Fetal liver endothelium, WNT5A, Organ-specific, Hematopoiesis

## Abstract

The human fetal liver is a critical organ for prenatal hematopoiesis, the study of which offers insights into niche signals that regulate the fates of hematopoietic stem and progenitor cells (HSPCs) during fetal development. Here, we demonstrate that human fetal liver endothelium uniquely supports the maturation and expansion of multilineage HSPCs. Specifically, co-culture of fetal liver-derived immature CD43^+^CD45^−^ hematopoietic cells with human fetal liver endothelial cells (ECs) led to a profound increase in the numbers of phenotypic CD45^+^CD34^+^ HSPCs and multilineage colony-forming progenitors generated in vitro, when compared to co-culture with ECs derived from other organs. We further identified a supportive role for EC-derived WNT5A in this process via gain- and loss-of-function studies within ECs. Our study emphasizes the importance of the organ-specific endothelial niche in supporting hematopoietic development and provides novel insight into signals that may facilitate HSPC expansion in vitro for clinical applications.

**Supplementary Information:**

The online version contains supplementary material available at 10.1186/s13287-021-02380-z.

## Introduction

The human fetal liver is a critical organ for prenatal hematopoiesis, where nascent hematopoietic stem and progenitor cells (HSPCs), generated from hemogenic endothelium in embryonic blood vessels, migrate to the fetal liver to further mature, proliferate, and differentiate into multilineage cells. During this process, the fetal liver provides critical niche signals to simultaneously generate blood cells that meet the immediate needs of the developing fetus and to massively expand the long-term hematopoietic stem cell pool, which sustains life-long hematopoiesis following migration to the bone marrow [1–5]. Previous studies have shown that adult hematopoiesis in the bone marrow requires support from the surrounding microenvironment, including the vasculature [3, 6–9]. Similarly, studies of murine fetal liver hematopoiesis have suggested a role for fetal liver stromal populations, including endothelial cells, in modulating HSPC fate [10–15]. However, few studies describe the endothelial-specific factors that promote HSPC expansion and maturation in the context of human development. Identifying the molecular mechanisms by which the fetal liver vasculature influences hematopoiesis could lead to techniques for improving the in vitro expansion of HSPCs for therapeutic applications [16]. Here, we sought to explore the contribution of the human fetal liver vascular niche that regulates the fates of HSPC. We demonstrated fetal liver vascular niche endothelial cells (ECs) uniquely support HSPC maturation and expansion compared with other organ-specific ECs, and that this support is partially dependent on EC-derived WNT5A. Altogether, our findings provide an enhanced understanding of the niche for human fetal liver hematopoiesis and lend new insights that may contribute to development of novel therapies for hematopoietic regeneration and disease modeling.

## Results and discussion

### The human fetal liver contains immature CD45^−^CD43^+^ hematopoietic cells that can mature to multilineage CD45^+^CD34^+^ HSPC

**Fig. 1:**
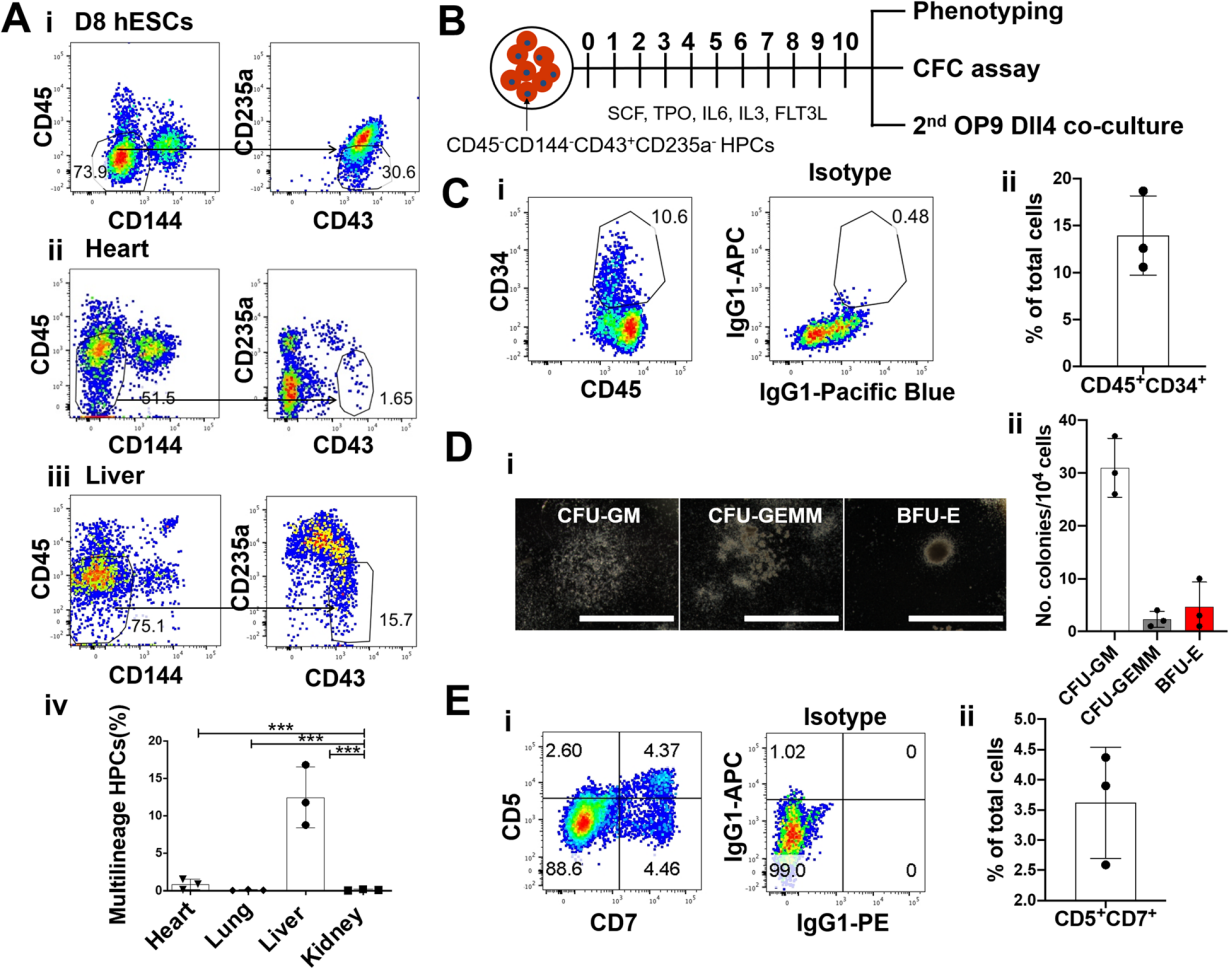
The human fetal liver contains immature CD45^−^CD43^+^ hematopoietic cells that can mature to multilineage CD45^+^CD34^+^ HSPC. **A** CD45^−^CD144^−^CD43^+^CD235a^−^ hematopoietic progenitor cells were detected at significantly higher frequency in the fetal liver than other fetal tissues. (i) CD45^−^CD144^−^CD43^+^CD235a^−^ cells were detected during in vitro human embryonic stem cell (hESC) differentiation (see also supplemental figure 1). (ii, iii) CD45^−^CD144^−^CD43^+^CD235a^−^ cells within the human fetal heart (ii) and liver (iii). (iv) Quantification of the percentage of CD45^−^CD144^−^CD43^+^CD235a^−^ HPCs among four fetal tissues (*n* = 3 donors, mean ± SEM, ***p < 0.001). **B** Schematic diagram of CD45^−^ CD144^−^CD43^+^CD235a^−^ cells sorted by FACS and cultured on subconfluent E4ORF1-transduced primary human umbilical vein endothelial cells (E4ECs) in serum-free media supplemented with hematopoietic cytokines for 10 days. **C**, **D** Following E4EC co-culture, CD45^−^CD144^−^CD43^+^CD235a^−^ cells generate (**C**, i) CD45^+^CD34^+^ cells as well as (**D**, i) colony-forming progenitors including CFU-GM, CFU-GEMM, and BFU-E (scale bar, 2000 μm). Quantification of CD45^+^CD34^+^cells (**C**, ii) and CFC assay (**D**, ii) (*n* = 3 donors mean ± SEM). **E** CD45^−^CD43^+^ HPCs from human fetal liver can differentiate into T lymphocytes. (i) Following primary culture of HPC on E4EC stroma, hematopoietic progeny was co-cultured with OP9-Dll4 for 14–21 days, and the induced non-adherent cells were characterized with early T cell markers CD5 and CD7 via flow cytometry. (ii) Quantification of CD5^+^CD7^+^ cells (*n* = 3 donors mean ± SEM)

To characterize hematopoietic populations in the human fetal liver, we performed flow cytometric analysis of cells from enzymatically digested human fetal liver tissues (16–20 weeks gestational age). In addition to expected CD45^+^CD34^+^ HSPCs (Figure S1A), we detected CD45^−^CD144^−^CD43^+^CD235a^−^ cells in the human fetal liver phenotypically comparable to early CD43^+^ hematopoietic progenitors identified during differentiation of human embryonic stem cells (hESCs) in vitro (Fig. 1A and Fig. S1B-C). As expected, these CD43^+^ hematopoietic progenitors were largely absent in other fetal organs that do not support fetal hematopoiesis. CD43 is a cell surface marker expressed on multilineage hematopoietic progenitors prior to expression of the pan-hematopoietic marker CD45 [17], suggesting the CD45^−^CD144^−^CD43^+^CD235a^−^ population may include immature hematopoietic progenitors that can give rise to mature multilineage CD45^+^CD34^+^ HSPC. To test this, we sorted human fetal liver CD45^−^CD144^−^CD43^+^CD235a^−^ cells by FACS (Fig. 1B) and examined their hematopoietic potential following co-culture on E4HUVEC (here after referred to as E4ECs) [18]. CD45^−^CD144^−^CD43^+^CD235a^−^ cells generated a population of CD45^+^ hematopoietic cells, including a subset of phenotypic CD45^+^CD34^+^ HSPC (Fig. 1C). Furthermore, the hematopoietic progeny following co-culture contained erythroid/myeloid lineage potential based on secondary colony-forming assays (Fig. 1D and Figure S1C-D) and lymphoid lineage potential based on production of CD5^+^CD7^+^ T lymphoid precursors following secondary OP9-Dll4 co-culture (Fig. 1E). These findings suggest that fetal liver CD43^+^CD45^−^ cells include immature hematopoietic precursors that can give rise to multilineage CD45^+^CD34^+^ HSPC.

### Fetal liver endothelium uniquely supports expansion of CD45^+^CD34^+^ cells and multilineage colony-forming progenitors from CD45^−^CD43^+^ cells

**Fig. 2:**
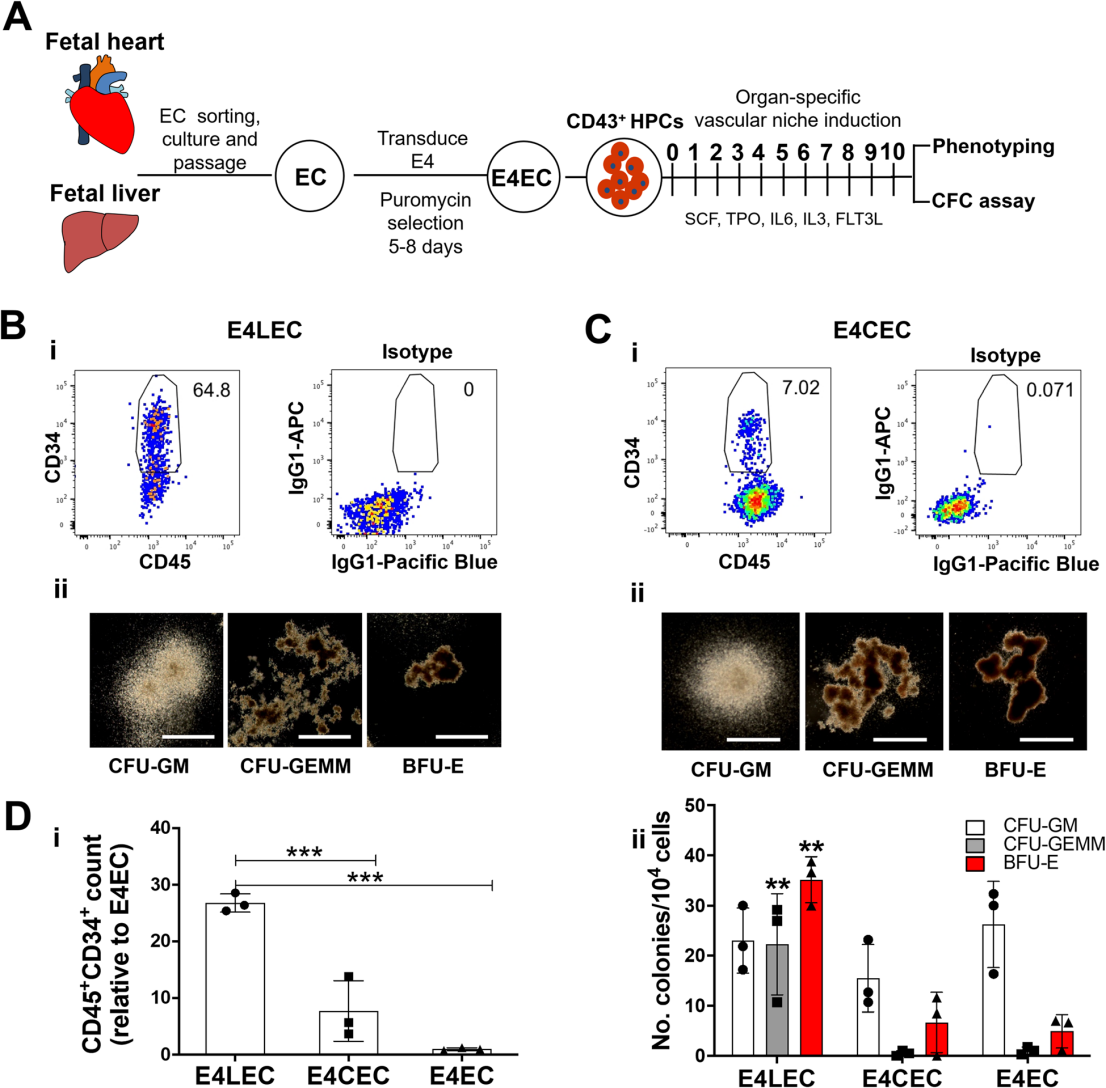
Fetal liver endothelium promotes expansion of CD45^+^CD34^+^ cells and multilineage colony-forming progenitors from CD45^−^CD43^+^ cells. **A** Schematic diagram of CD45^−^CD43^+^ HPCs co-culture with human E4 cardiac endothelial cells (E4CEC) and E4 liver endothelial cells (E4LEC). E4CEC and E4LEC were then separately co-cultured with CD45^−^CD144^−^CD43^+^ fetal liver cells for 10 days. The generated non-adherent cells were collected for phenotypic characterization and colony formation assays. **B**, **C** Phenotypic characterization (i) and hematopoietic colony formation (ii) following 10 days of co-culture with E4LEC (**B**) and E4CEC (**C**), respectively. Scale bar, 1000 m. **D** Percentages of CD45^+^CD34^+^ cells assessed by flow cytometry and total cell counts at day 10 were multiplied to quantify absolute numbers of cells at day 10. Bar graph in (i) represents the fold changes of CD45^+^CD34^+^ cells numbers cultured on E4LECs and ECECs relative to E4ECs (*n* = 3 donors, mean ± SEM, ***p ≤ 0.001). Quantification of (ii) hematopoietic colony-forming progenitors derived from HPCs after co-cultured with different ECs (*n* = 3 donors, mean ± SEM, **p ≤ 0.01)

We have previously demonstrated that human fetal endothelial cells can retain organ-specificity after isolation and passaging, making it possible to explore the endothelial niche factors under in vitro co-culture conditions [19]. Here, we further engineered these ECs to allow for their growth in serum-free hematopoietic co-culture conditions. Specifically, we generated E4ORF1-transduced liver ECs (E4LECs) and cardiac ECs (E4CEC) from the same fetal donor (Fig. 2A and Figure S2A) [18, 20–22]. By quantifying several liver-specific transcripts (i.e., *HOXB4*, *HOXB7*) [19], for both primary ECs and E4ECs (Figure S2B), we demonstrated these engineered cell lines maintained their organ-specific gene expression.

We next compared the in vitro generation of multilineage hematopoietic progenitors from fetal liver CD45^−^CD43^+^CD144^−^ cells when co-cultured with E4LECs, E4ECs, or E4CECs. CD45^−^CD43^+^CD144^−^ cells co-cultured with E4LECs generated significantly more phenotypic CD45^+^CD34^+^ HSPC compared with E4EC and E4CEC co-culture (Fig. 2Bi–Di), as well as higher numbers of colony-forming progenitors (Fig. 2Bii–Dii). These findings demonstrate the fetal liver EC niche optimally supports fetal liver HSPC maturation and expansion.

### WNT5A is an important fetal liver-specific vascular niche factor that promotes hematopoiesis

**Fig. 3:**
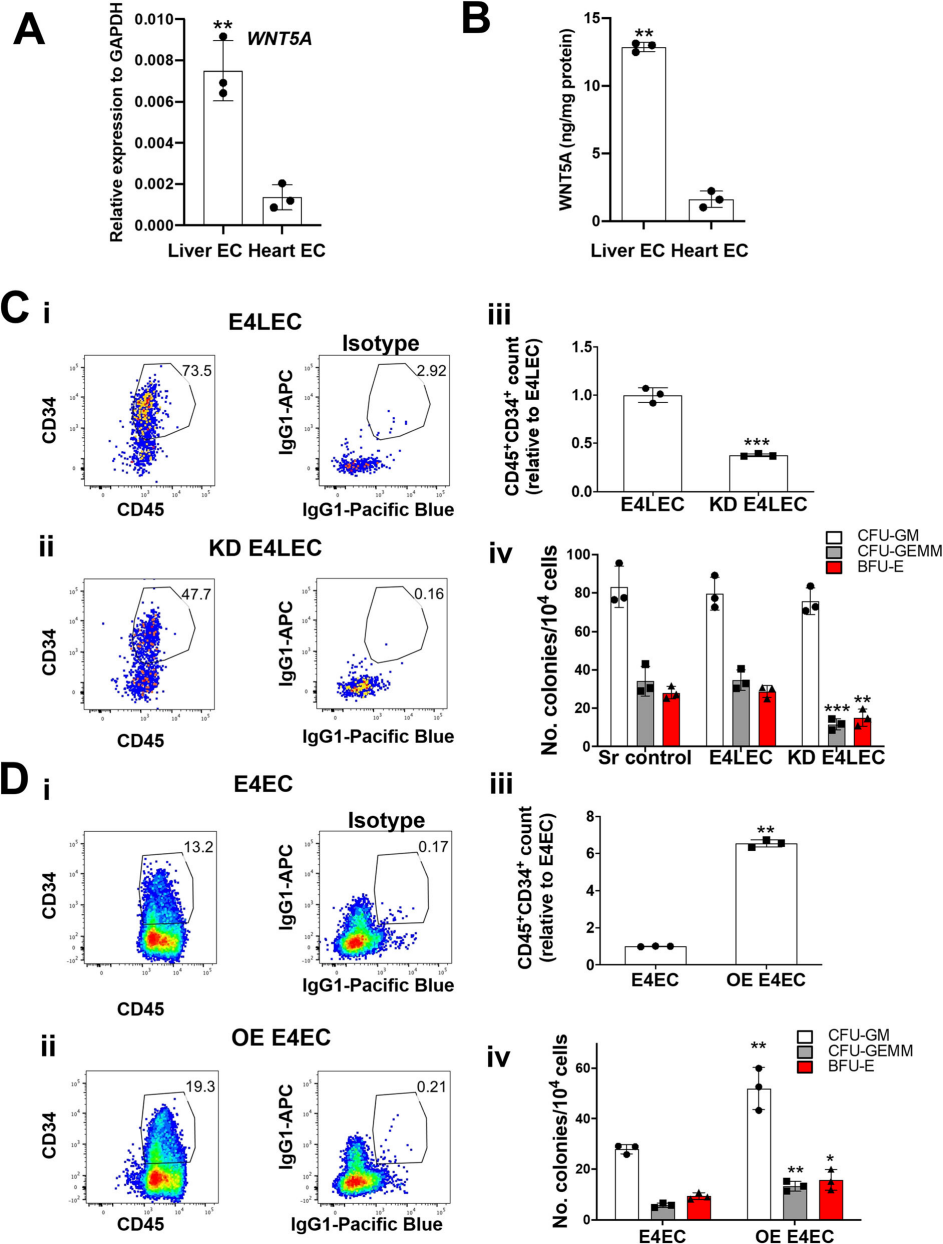
WNT5A is highly expressed by the liver endothelial niche and promotes CD45^+^CD34^+^ cell generation in vitro. **A** RT-qPCR of *WNT5A* expression (*n* = 3 donor sets, mean ±SEM, **p ≤ 0.01). **B** Quantitative measurement of WNT5A protein via ELISA assays (*n* = 3 donor sets, mean ± SEM, **p ≤ 0.01). **C** Knockdown of *WNT5A* in human fetal liver ECs decreases the number of CD45^+^CD34^+^ cells and colony-forming progenitors generated following co-culture with human fetal liver CD45^−^CD43^+^ HPCs. (i, ii) Flow cytometric analysis of CD45^+^CD34^+^ cells generated from HPCs following 10 days of co-culture on E4LEC (i) or KD-WNT5A E4LEC (ii). (iii) Quantification of CD45^+^CD34^+^ cells produced following co-culture, normalized to those produced from co-culture with E4LECs (*n* = 3 donors, mean ± SEM, ***p ≤ 0.0001). (iv) Quantification of colony-forming progenitors following co-culture on E4LEC, KD-WNT5A E4LEC, or scrambled control (sr) E4LEC (*n* = 3 biological replicates, mean ± SEM, **p ≤ 0.01, ***p ≤ 0.001). **D** Overexpression of WNT5A in E4ECs increased the number of CD45^+^CD34^+^ cells and colony-forming progenitors generated following co-culture with human fetal liver CD45^−^CD43^+^ HPCs. (i, ii) Flow cytometric analysis of CD45^+^CD34^+^ cells generated from HPC following 10 days co-culture on E4ECs (i) or OE-WNT5A E4EC (ii). (iii) Quantification of CD45^+^CD34^+^ cell numbers (*n* = 3 donors, mean ± SEM, **p ≤ 0.01) and (iv) colony-forming progenitors (*n* = 3 donors, mean ± SEM, * *p* ≤ 0.05, ** *p* ≤ 0.01)

We have previously shown that fetal liver ECs are highly enriched for pathways involved in regulation of hematopoietic cell proliferation and bone marrow development in comparison to fetal cardiac ECs [19]. Among the differentially expressed genes, WNT5A, a member of the non-canonical Wnt pathway demonstrated to have a role in regulating hematopoiesis [23], is highly upregulated (16-fold higher) in human fetal liver ECs compared to cardiac ECs [19]. Using RT-qPCR and enzyme-linked immunosorbent assay (ELISA), we validated that *WNT5A* transcript and protein expression are significantly higher in fetal liver ECs compared to cardiac ECs (Fig. 3A and B). Given the established role for non-canonical WNT5A in promoting HSPC self-renewal and repopulation [23], we hypothesized that WNT5A plays an influential role within the fetal liver endothelial niche to support HSPC maturation/expansion. To test the function of WNT5A, we generated E4LECs with endogenous *WNT5A* knocked down by small hairpin RNA (shRNA) (Figure S3A and B). RT-qPCR confirmed significant suppression of *WNT5A* in knockdown E4LECs (KD E4LECs) compared with E4LECs (Figure S3Bi). We then compared the capacity of KD E4LECs versus control wild type E4LECs to support generation of HSPC from fetal liver CD45^−^CD43^+^ cells. CD45^−^CD43^+^ cells co-cultured with KD E4LECs generated significantly fewer CD45^+^CD34^+^ phenotypic HSPC (3.0 ± 0.3-fold reduction) compared with those co-cultured on wild type E4LECs (Fig. 3Ci–iii). Furthermore, CD45^−^CD43^+^ cells co-cultured with KD E4LECs generated decreased numbers of colony-forming progenitors, particularly CFU-GEMM (3.8 ± 0.9-fold reduction) and BFU-E (2.0 ± 0.7-fold reduction) (Fig. 3Civ). Altogether, these findings indicate that knockdown of WNT5A significantly impairs the ability of E4LECs to support HSPC maturation and multilineage CFU progenitor formation/expansion in vitro.

To determine whether WNT5A overexpression could enhance the ability of non-liver endothelial cells to support the maturation/expansion of CD43^+^CD45^−^ cells into phenotypic CD45^+^CD34^+^ HSPC, we generated an E4EC line that overexpressed WNT5A (OE E4ECs). OE E4ECs exhibited 6.9 ± 2.8-fold higher *WNT5A* transcript expression compared to control E4ECs by RT-qPCR (Figure S3Ci). Co-culture of fetal liver CD45^−^CD43^+^ cells with OE E4ECs led to an increase in the number of phenotypic CD45^+^CD34^+^ HSPCs produced (6.6 ± 0.4-fold increase) compared to co-culture with E4ECs (Fig. 3Di–iii). Moreover, CD45^−^CD43^+^ cells co-cultured with OE E4ECs showed an improved yield of CFU-GM (1.9 ± 0.3-fold increase), CFU-GEMM (2.3 ± 0.01-fold increase) and BFU-E (1.7 ± 0.5-fold increase) compared to CD45^−^CD43^+^ cells co-cultured with E4ECs (Fig. 3Div). Taken together, this data demonstrates that augmented WNT5A expression in non-liver ECs enhances their ability to support hematopoiesis and promote HSPC maturation/expansion.

### WNT5A acts in a cell autonomous manner in the fetal liver niche to promote hematopoiesis

WNT5A has been shown to promote endothelial proliferation and survival [24]; it is therefore conceivable that manipulation of WNT5A here influenced both HSPC differentiation and liver EC function. Thus, we sought to further investigate whether or not the role we observed for WNT5A on HSPC differentiation was cell autonomous. To do this, we first examined the impact of WNT5A on liver EC function. We previously showed that WNT5A expression is significantly increased in fetal liver ECs compared to cardiac ECs, though cardiac ECs exhibit significantly higher angiogenic and metabolic properties [19]. To clarify the impact of WNT5A on LECs, we compared the expression of several genes related to proliferation, angiogenesis, and quiescence in E4LECs and KD E4LECs via RT-qPCR and observed no significant difference in expression of selected genes (Figure S3D). To further explore a possible impact of WNT5A on liver EC function, we cultured E4ECs in soluble WNT5A or E4LECs in WNT5A neutralizing antibodies. Soluble WNT5A appeared to enhance junctional expression of VE-cadherin and cortical organization of actin cytoskeleton (Figure S4A and B). However, there was no significant difference in cell proliferation or transcript abundance of selected genes (*MMP1*, *TEK*, *PCNA*, *NOS3,* etc.) (Figure S4C and D).

Next, we assessed the impact of soluble WNT5A on HSPC differentiation in the absence of ECs. Briefly, CD43^+^ immature hematopoietic cells were cultured in stromal-free conditions in the presence or absence of 250 ng/mL soluble WNT5A for 3 days. We observed the presence of soluble WNT5A significantly increased production of a CD34^+^CD45^+^ HSPC population compared to the control (Figure S4E and F). Moreover, CFU-GEMM formation was only observed when soluble WNT5A was added to the stromal-free culture (Figure S4G). In summary, these findings demonstrate that modulation of WNT5A expression has a minimal impact on fetal liver EC function, while the presence of soluble WNT5A in stromal-free conditions is sufficient to enhance HSPC maturation/expansion. Not unexpectedly, the presence of soluble WNT5A in stromal-free conditions did not fully recapitulate the robust quantitative output of phenotypic HSPC or colony-forming progenitors generated by co-culture on fetal liver ECs. Likewise, overexpression of WNT5A in non-liver ECs was not sufficient to promote quantitatively equivalent generation of phenotypic HSPC compared to E4LECs. Both observations indicate that, although WNT5A may be a key factor, there are likely other factors uniquely produced by the fetal liver endothelium that contribute to the support of definitive HSPC maturation and expansion. Thus, it will be important for further studies to identify other niche factors which support fetal liver hematopoiesis, possibly in concert with WNT5A.

Taken together, the data presented here suggests that WNT5A likely regulates hematopoiesis in the fetal liver niche in a cell autonomous manner. Indeed, several previous studies have indicated WNT5A acts in a similar fashion in the adult bone marrow vascular niche. For example, WNT5A interacts with the Ryk protein, a Wnt ligand receptor, to promote HSC quiescence and enhance repopulation via suppression of reactive oxygen species, which are known to promote HSC proliferation [25]. Additionally, WNT5A has been shown to suppress canonical Wnt3a-mediated effects on cells, via the destabilization of β-catenin, in order to promote quiescence in adult HSCs [23]. Beyond cell cycle, WNT5A is also able to influence the actin cytoskeleton of adult HSCs through ZEB1-assocaited genes involved in the actin polymerization pathway. Aberrant regulation of this pathway, due to partial loss of WNT5A expression in the bone marrow niche, results in adhesion and homing defects for HSCs in vivo and leads to increased differentiation in vitro [26]. In total, these studies provide support for the notion that WNT5A is able to regulate hematopoiesis in a cell autonomous manner. Identifying possible molecular mechanisms, future studies could further explore how WNT5A contributes to HSPC maturation and expansion in the context of the fetal liver vascular niche.

In summary, our findings improve our understanding of how the human fetal liver niche supports hematopoiesis and shed light into human hematopoietic development that could be used for in vitro production of definitive HSPC, such as from human pluripotent stem cells, for therapeutic applications. Combined with our previous work, our studies support the concept that organ-specific ECs have unique transcriptomes and cellular functions that support organ-specific aspects of development [19]. Future studies could identify other fetal liver vascular-specific paracrine signals, as well as different endothelial subpopulations and other fetal liver cell types that contribute to the formation of the fetal liver niche in supporting HSPC differentiation, self-renewal, and proliferative expansion.

## Materials and methods

Flow cytometry analysis for cell populations in human fetal liver: All experiments involving human fetal organs were approved by the Institutional Review Board of the University of Washington (IRB447773EA). Human fetal organs including the liver, kidney, heart, and lungs were obtained from abortion material (age 16-20 weeks) upon informed consent. Tissue was finely minced in serum-free EBM-2 endothelial growth medium (Lonza) supplemented with 1 mg/mL collagenase type IV (Sigma) and 100 U/ml DNase (Roche) and incubated for 30 min at 37 0C in a water bath with shaking. The resulting tissue homogenate was filtered through a 40 μm cell strainer (BD Falcon) to remove tissue debris and large vessels. Enzymatically digested liver populations were stained for CD45, CD34, CD144, CD43, and CD235a, and corresponding isotype controls, as indicated in Table S1). Multiparameter FACS analysis was performed on a BD FACSCanto II (Beckton Dickinson) and quantitated with FlowJo software (Tree Star).

Generation of hESC-derived hematopoietic progenitors: hESC differentiation was performed as previously described [27]. In brief, hESC colonies were dissociated with Trypsin-Versene (Life Technologies) and resuspended in pluripotency medium containing CHIR99021(Cayman Chemical) for 24 h. Cells were then cultured with RPMI medium (Life Technologies) supplemented with 50 ng ml−1 activin A (R&D Systems) and 40 ng ml−1 BMP4 (R&D Systems) on days 0 and 1, respectively. On day 2, directed endothelial differentiation was performed by replacing the media with Stempro34 media (Life Technologies) containing 200 ng ml−1VEGF (PeproTech), 10 ng ml−1 BMP4 (R&D Systems), 5 ng ml−1 bFGF (PeproTech), 50 μg ml−1ascorbic acid (Sigma-Aldrich), and 0.4 mM monothioglycerol (Sigma-Aldrich) for 5 days. From days 5-8, medium was changed to StemPro-34 with 10 ng ml−1 bFGF, 15 ng ml−1VEGF, 10 ng ml−1 interleukin (IL)-6 (PeproTech), 25 ng ml−1 IGF-1 (PeproTech), 5 ng ml−1 IL-11 (PeproTech), and 50 ng ml−1 SCF (PeproTech). On day 8, freshly dissociated hESCs were stained with multilineage HPCs markers.

E4ORF1 transduction of fetal liver and cardiac ECs: MSCV-N E4orf1 was obtained as a gift from Karl Munger (Addgene plasmid # 38063). For retroviralvector production, platinum GP retroviral packaging cells (Cell Biolabs) were cultured in 150 mm dishes (Corning Life Science) to 80-90 % confluency, and transfected with VSV-G, gag, pol, E4orf1, and Lipofectamine 3000 (Invitrogen) in DMEM (Thermo Fisher Scientific) for 17 h. These packaging cells were then cultured in DMEM supplemented with HEPES (Thermo Fisher Scientific) for 48h and supernatant was collected, filtered, and concentrated via ultracentrifugation. The viral pellet was resuspended in serum-free DMEM and stored at -80°C.

Human fetal liver and cardiac ECs were isolated and cultured from the same organ source as the hematopoietic progenitor cells, using the methods described previously [19]. At passage 2, both types of cells were plated at 70 - 80% confluency in separate culture plates, and incubated twice with viral particles at 12-hour intervals. After infection, cells were selected by 2.5 μg/ml Puromycin (Invitrogen) for 5-8 days. E4ORF1 transduction was verified by RT-qPCR. The ability of transduced cell lines to survive without serum was verified.

RNA isolation and reverse-transcription quantitative PCR (RT-qPCR): Total RNA from human fetal liver and cardiac endothelial cells was purified usingthe RNAeasy Mini Kit (Qiagen). Residual DNA was removed by on-column DNase digestion. RT-qPCR was performed using the Real-time PCR System(Applied Biosystems) with Fast SYBR Green Master Mix (Applied Biosystems). The amount of target RNA gene was normalized to GAPDH RNA. Primer sequences were as follows: WNT5A F-5’- TAG CAG CAT CAG TCC ACA AA -3’ and R-5’- CAA AAC ACG GCA TCT CTC TT -3’, E4ORF1 F-5’-CCT GCG GGT ATG TAT TCC CC-3’ and R-5’- GAC AGC TCC TCG GTC ATG TC-3’

Detection of WNT5A protein by ELISA: The total protein concentrations of liver and cardiac EC lysates were quantified using Pierce™ BCA (Thermo Fisher Scientific). ELISA assay for WNT5A quantification was performed using a commercially available ELISA kit (LS Bio) according to the manufacturer’s instructions.

KD E4LEC and OE E4EC production: pGIPZ lentiviral shRNA against WNT5A gene (Dharmacon) and scrambled sequence control constructs encoding puromycin resistance and GFP were used. Lentiviral particles were generated in HEK293T cells using a second-generation packaging system. E4LECs were transfected with shRNA and scramble controls. Transduced cells were selected by 2.5 μg/ml Puromycin (Invitrogen) for 5-8 days and GFP-positive KD E4LECs were sorted by flow cytometry and transgene expression levels were quantified by RT-qPCR. pLX304 lentiviral vector (Dharmacon) that encodes a blasticidin resistance genes and WNT5A ORF expression from a cytomegalovirus (CMV) promoter was used to generate WNT5A overexpressed HUVECs. WNT5A lentivirus was produced in HEK293T cells and used to infect HUVECs. After infection, cells were selected by 2.5 μg/ml blasticidin (Invitrogen) for 5-8 days. WNT5A transduction was verified by RT-qPCR.

Cell culture and colony forming assays: CD45-CD144-CD43+ CD235a- cells from human fetal livers were sorted by FACS and cultured on E4orf1-transduced HUVECs (E4ECs), E4orf1-transduced liver ECs (E4LECs), E4orf1-transduced cardiac ECs (E4CECs), KD E4LEC and OE E4EC, as indicated, in serum-free StemSpan SFEM (StemCell Technologies) supplemented with Pen-Strep (Invitrogen), 100 μM monothioglycerol (MTG; Sigma-Aldrich), 50 μg/ml ascorbic acid, 50 ng/ml rhSCF (Peprotech), 20 ng/ml rhTPO (Peprotech), 20 ng/ml rhIL6 (Peprotech), 20 ng/ml rh IL3 (Peprotech), and 20 ng/ml rhFLT3L (Peprotech) for 10 days. Half the media was replaced with fresh media on day 3. Following 10 days of co-culture, non-adherent cells were resuspended by vigorous pipetting and passed through a 40 μm cell strainer (BD Falcon). The harvested cells were either analyzed by flow cytometry for phenotypic characterization or selected for CD34-positive cells via MACS for colony formation assays. The selected CD34+ populations were plated in complete methylcellulose medium containing human cytokines including SCF, GM-CSF, IL-3, G-CSF, EPO (Stem cell technologies), and supplemented with 20 ng/mL rhFLT3L (Peprotech) and 50ng/mL IL-6 (Peprotech). The plates were imaged, and colonies were analyzed at day 14 for total colony number, and each colony classified based upon morphologic criteria as granulocyte, erythrocyte, monocyte, megakaryocyte (CFU-GEMM), granulocyte/monocyte/macrophage (CFU-GM) or burst-forming erythroid (BFU-E). Colonies were stained with Giemsa using a Hematek II slide stainer following cytospin at 300 x g for 10 min and imaged on a Hamamatsu Nanozoomer Digital Pathology system.

T cell differentiation: After co-culture with endothelial cells, non-adherent hematopoietic cells were resuspended by vigorous pipetting and passed through a 40 μm cell strainer (BD Falcon). The harvested cells were plated on bone marrow stromal cell line expressing notch ligand Delta-like 4 (OP9-Dll4) in α-MEM (Invitrogen) supplement with Pen-Strep (Invitrogen), 20% FBS (Hyclone), 100 μM monothioglycerol (MTG; Sigma-Aldrich), 50 μg/ml ascorbic acid, 50 ng/ml rhSCF (Peprotech), 20 ng/ml rhIL7 (Peprotech), and 20 ng/ml rhFLT3L (Peprotech) for 14-21 days. Floating hematopoietic cells were collected and stained with CD5 and CD7 for flow analysis to confirm the presence of T cells.

Stromal free culture conditions: hESC differentiation was performed as described above in ‘Generation of hESC-derived hematopoietic progenitors’ up to day 5. On day 5, wells were harvested and co-cultured with OP9 cells in StemPro-34 supplemented with 100 μM MTG (Sigma-Aldrich), 50 μg/ml ascorbic acid, 50 ng/ml rhSCF (Peprotech), 20 ng/ml rhTPO (Peprotech), 20 ng/ml rh IL6 (Peprotech), 20 ng/ml rh IL3 (Peprotech), and 20 ng/ml rh FLT3L (Peprotech) for 4 days. Cells were then passaged to stromal free conditions on retronectin (5 μg/ml; Takara Bio) coated wells in serum-free StemSpan SFEM (StemCell Technologies) supplemented with 50 ng/ml rhSCF (Peprotech), 20 ng/ml rhTPO (Peprotech), 20 ng/ml rhIL6 (Peprotech), 20 ng/ml rh IL3 (Peprotech), and 20 ng/ml rhFLT3L (Peprotech) plus/minus 250 ng/ml rh Wnt5a (R&D Systems). Following 3 days of stromal free culture, cells were harvested for FACS analysis or CFU assay as described above.

Statistical analysis: All data are presented as the mean ± error of the mean (SEM), and the results were analyzed using Prism Software (GraphPad, USA). A two-tailed Student’s t test was used to compare two groups. The one-way analysis of variance (ANOVA) with a Tukey’s post-hoc test was used to compare more than two groups.

## Supplementary Information

**Additional file 1: Table S1.** Antibodies used in this study. **Figure S1. A**. Phenotypic hematopoietic stem and progenitor cells (HSPCs, CD45^+^CD34^+^) were found to represent 5.4 ± 0.4 % (*n* = 3 donors, mean ± SEM) of total liver cells. **B**. Representative flow cytometry profiles of (i) hESC- HPCs with isotype controls, (ii-v) fresh total cell tissue suspension from fetal human kidney(ii), heart(iii), lung(iv) and liver(v) stained with markers for HPCs. **C**. Multiple hematopoietic cell types were identified including (a) polychromatic normoblasts, (b) orthochromic normoblasts, (c) polychromatic erythrocytes, (d) promyelocytes, (e) myelocytes, (f) metamyelocytes (g) polymorphonuclear neutrophils, (h) eosinophils, (i) monocytes, (j) basophils, and (k) macrophages (scale bar: 10 m). **D**. Flow cytometry profiles of the colonies stained with erythroid and myeloid markers. **Figure S2. A**. Bright field image of E4ORF1 transduced liver ECs (i) and heart ECs (ii) cultured with and without serum media. Scale bar: 200 m. (iii) Ectopic expression of E4ORF1 in liver ECs (E4LECs), HUVECs (E4ECs), and cardiac ECs (E4CECs) compared with E4ECs was confirmed using a reverse-transcription quantitative PCR (RT-qPCR) ( *n* =3 donors, mean ± SEM) **B**. Comparison of mRNA expression of selected genes for primary and E4ORF1 transfected heart and liver endothelial cells for liver endo-specific genes. (*n* = 3, mean ± SEM, * *p* ≤ 0.05, **p ≤ 0.01, ***p ≤ 0.001). **Figure S3**. E4LECs were transduced with lentiviral vectors containing WNT5A (WNT5A-IRES-GFP) under the control of CMV promoter (i) FACS sorting scheme for GFP positive cells. (ii) Representative images of sorted GFP^+^ E4LECs. Scale bar: 0.5 mm. **B**. Knockdown of *WNT5A* on E4LECs. (i) RT-qPCR confirms *WNT5A* knockdown on E4LECs after transduction with pGIPZ lentiviral shRNA against *WNT5A* (*n* = 3 donors, mean ±SEM, * *p* ≤ 0.05) (ii) Schematic diagram for preparation of KD E4LECs and co-culture with liver HPCs for 10 days. **C**. Overexpression of *WNT5A* on E4ECs. (i)RT-qPCR confirms that *WNT5A* is overexpressed in E4ECs transduced with lentiviral vector that encodes blasticidin resistance genes and *WNT5A* gene. (*n* = 3, mean ± SEM, * *p* ≤ 0.05) (ii) Schematic diagram of E4ECs co-cultured with liver HPCs for 10 days. **D**. RT-qPCR showed that *WNT5A* knockdown on E4LECs led to non-significant changes in EC gene expression including *PCNA*, *DLL4*, *MMP10*, *NOS3* and *VEGFR2* (*n* = 3 donors, mean ±SEM). **Figure S4.** The role of soluble WNT5A on ECs and HPCs. **A-B**. Immunostaining of E4ECs culture at 0 or 100ng/mL WNT5A (A) and E4LECs at 0 or 10 ng/mL neutralization antibody for WNT5A (B) for 24 hours. Red: VE-Cad, and Magenta: F-actin. **C-D**. Quantification of Ki67+ cells (C) and expression of genes (D) for both ECs before and after respective treatments. **E-F**. Phenotyping (E-F) of stromal free culture of CD43^+^CD45^-^CD144^-^ cells with or without WNT5A and CFU analysis (G) after 3 days of culture shows significant increase of CD45^+^CD34^+^ cells, and appearance of CFU-GEMM with soluble WNT5A (*n* = 3-6, mean ± SEM, * *p* ≤ 0.05).

## Data Availability

All data needed to evaluate the conclusions in this paper are present in the paper and the Supplementary Materials. Additional data related to this paper may be requested from the corresponding author (yingzy@uw.edu).

## Abbreviations

HSPCs: Hematopoietic stem and progenitor cells
ECs: Endothelial cells
CD: Cluster of differentiation
WNT5A: Wnt (Wingless-related integration site) Family Member 5A
E4ECs: E4ORF1 transduced HUVECs (human umbilical vein ECs)
E4LECs: E4ORF1 transduced liver ECs
E4CECs: E4ORF1 transduced cardiac ECs
ELISA: Enzyme-linked immunosorbent assay
RT-qPCR: Quantitative reverse-transcription PCR
CFU-GEMM: Colony-forming unit-granulocyte, erythroid, macrophage, megakaryocyte
BFU-E: Erythroid burst-forming units
CFU-GM: Colony-forming unit for granulocytes and macrophages
hESCs: Human embryonic stem cells
