# Supplementary material for: WNT5A from the fetal liver vascular niche supports human fetal liver hematopoiesis
Source: Stem Cell Res Ther. 2021 Jun 5;12:321. doi: 10.1186/s13287-021-02380-z (PMC8180064; doi:10.1186/s13287-021-02380-z)
Supplement: Supplementary file 1 — Additional file 1: Table S1. Antibodies used in this study. Figure S1. A. Phenotypic hematopoietic stem and progenitor cells (HSPCs, CD45+CD34+) were found to represent 5.4 ± 0.4 % (n = 3 donors, mean ± SEM) of total liver cells. B. Representative flow cytometry profiles of (i) hESC- HPCs with isotype controls, (ii-v) fresh total cell tissue suspension from fetal human kidney(ii), heart(iii), lung(iv) and liver(v) stained with markers for HPCs. C. Multiple hematopoietic cell types were identified including (a) polychromatic normoblasts, (b) orthochromic normoblasts, (c) polychromatic erythrocytes, (d) promyelocytes, (e) myelocytes, (f) metamyelocytes (g) polymorphonuclear neutrophils, (h) eosinophils, (i) monocytes, (j) basophils, and (k) macrophages (scale bar: 10 m). D. Flow cytometry profiles of the colonies stained with erythroid and myeloid markers. Figure S2. A. Bright field image of E4ORF1 transduced liver ECs (i) and heart ECs (ii) cultured with and without serum media. Scale bar: 200 m. (iii) Ectopic expression of E4ORF1 in liver ECs (E4LECs), HUVECs (E4ECs), and cardiac ECs (E4CECs) compared with E4ECs was confirmed using a reverse-transcription quantitative PCR (RT-qPCR) ( n =3 donors, mean ± SEM) B. Comparison of mRNA expression of selected genes for primary and E4ORF1 transfected heart and liver endothelial cells for liver endo-specific genes. (n = 3, mean ± SEM, * p ≤ 0.05, **p ≤ 0.01, ***p ≤ 0.001). Figure S3. E4LECs were transduced with lentiviral vectors containing WNT5A (WNT5A-IRES-GFP) under the control of CMV promoter (i) FACS sorting scheme for GFP positive cells. (ii) Representative images of sorted GFP+ E4LECs. Scale bar: 0.5 mm. B. Knockdown of WNT5A on E4LECs. (i) RT-qPCR confirms WNT5A knockdown on E4LECs after transduction with pGIPZ lentiviral shRNA against WNT5A (n = 3 donors, mean ±SEM, * p ≤ 0.05) (ii) Schematic diagram for preparation of KD E4LECs and co-culture with liver HPCs for 10 days. C. Overexpression of WNT5A on E4ECs. (i)RT-q [file 13287_2021_2380_MOESM1_ESM.docx]

**SUPPLEMENTAL MATERIALS**

Table S1. Antibodies used in this study

| Antigen | Fluorochrome | Source | Catalogue number |
| --- | --- | --- | --- |
| CD45 | Pacific blue | BioLegend | 304022 |
| CD45 | APC-Cy™7 | BD Biosciences | 348795 |
| CD43 | APC | BD Biosciences | 560198 |
| CD43 | Pacific blue | BD Biosciences | 744813 |
| CD144 | PerCP-Cy^TM^5.5 | BD Biosciences | 561566 |
| CD34 | FITC | BD Biosciences | 555821 |
| CD34 | APC | BD Biosciences | 555824 |
| CD235a | PE | Thermo Fisher Scientific | 12-9987-80 |
| CD235a | PE/Cy7 | BioLegend | 349111 |
| CD71 | PE | BD Biosciences | 555537 |
| CD11b | PE | BD Pharmingen | 555388 |
| CD5 | APC | BioLegend | 300611 |
| CD7 | PE | BioLegend | 343105 |
| Mouse IgG | Pacific blue | BioLegend | 400131 |
| Mouse IgG | PE | BD Biosciences | 555749 |
| Mouse IgG | APC | BD Pharmingen | 555751 |
| Mouse IgG | FITC | BD Biosciences | 555748 |
| Mouse IgG | PerCP-Cyanine5.5 | eBioescience | 45-4724-80 |

**SUPPLEMENTARY DATA**

***Flow cytometry analysis for cell populations in human fetal liver.*** All experiments involving human fetal organs were approved by the Institutional Review Board of the University of Washington (IRB447773EA). Human fetal organs including the liver, kidney, heart, and lungs were obtained from abortion material (age 16-20 weeks) upon informed consent. Tissue was finely minced in serum-free EBM-2 endothelial growth medium (Lonza) supplemented with 1 mg/mL collagenase type IV (Sigma) and 100 U/ml DNase (Roche) and incubated for 30 min at 37 ^0^C in a water bath with shaking. The resulting tissue homogenate was filtered through a 40 μm cell strainer (BD Falcon) to remove tissue debris and large vessels. Enzymatically digested liver populations were stained for CD45, CD34, CD144, CD43, and CD235a, and corresponding isotype controls, as indicated in Table S1). Multiparameter FACS analysis was performed on a BD FACSCanto II (Beckton Dickinson) and quantitated with FlowJo software (Tree Star).

***Generation of hESC-derived hematopoietic progenitors.*** hESC differentiation was performed as previously described.[[27](#_ENREF_27)] In brief, hESC colonies were dissociated with Trypsin-Versene (Life Technologies) and resuspended in pluripotency medium containing CHIR99021(Cayman Chemical) for 24 h. Cells were then cultured with RPMI medium (Life Technologies) supplemented with 50 ng ml^−1^ activin A (R&D Systems) and 40 ng ml^−1^ BMP4 (R&D Systems) on days 0 and 1, respectively. On day 2, directed endothelial differentiation was performed by replacing the media with Stempro34 media (Life Technologies) containing 200 ng ml^−1^VEGF (PeproTech), 10 ng ml^−1^ BMP4 (R&D Systems), 5 ng ml^−1^ bFGF (PeproTech), 50 μg ml^−1^ascorbic acid (Sigma-Aldrich), and 0.4 mM monothioglycerol (Sigma-Aldrich) for 5 days. From days 5-8, medium was changed to StemPro-34 with 10 ng ml^−1^ bFGF, 15 ng ml^−1^VEGF, 10 ng ml^−1^ interleukin (IL)-6 (PeproTech), 25 ng ml^−1^ IGF-1 (PeproTech), 5 ng ml^−1^ IL-11 (PeproTech), and 50 ng ml^−1^ SCF (PeproTech). On day 8, freshly dissociated hESCs were stained with multilineage HPCs markers.

***E4ORF1 transduction of fetal liver and cardiac ECs.*** MSCV-N E4orf1 was obtained as a gift from Karl Munger (Addgene plasmid # 38063). For retroviral vector production, platinum GP retroviral packaging cells (Cell Biolabs) were cultured in 150 mm dishes (Corning Life Science) to 80-90 % confluency, and transfected with VSV-G, gag, pol, E4orf1, and Lipofectamine 3000 (Invitrogen) in DMEM (Thermo Fisher Scientific) for 17 h. These packaging cells were then cultured in DMEM supplemented with HEPES (Thermo Fisher Scientific) for 48h and supernatant was collected, filtered, and concentrated via ultracentrifugation. The viral pellet was resuspended in serum-free DMEM and stored at -80°C.

Human fetal liver and cardiac ECs were isolated and cultured from the same organ source as the hematopoietic progenitor cells, using the methods described previously. [19] At passage 2, both types of cells were plated at 70 - 80% confluency in separate culture plates, and incubated twice with viral particles at 12-hour intervals. After infection, cells were selected by 2.5 µg/ml Puromycin (Invitrogen) for 5-8 days. E4ORF1 transduction was verified by RT-qPCR. The ability of transduced cell lines to survive without serum was verified.

***RNA isolation and reverse-transcription quantitative PCR (RT-qPCR)****.* Total RNA from human fetal liver and cardiac endothelial cells was purified using the RNAeasy Mini Kit (Qiagen). Residual DNA was removed by on-column DNase digestion. RT-qPCR was performed using the Real-time PCR System (Applied Biosystems) with Fast SYBR Green Master Mix (Applied Biosystems). The amount of target RNA gene was normalized to GAPDH RNA. Primer sequences were as follows: WNT5A F-5’- TAG CAG CAT CAG TCC ACA AA -3’ and R-5’- CAA AAC ACG GCA TCT CTC TT -3’, E4ORF1 F-5’- CCT GCG GGT ATG TAT TCC CC-3’ and R-5’- GAC AGC TCC TCG GTC ATG TC-3’

***Detection of WNT5A protein by ELISA.*** The total protein concentrations of liver and cardiac EC lysates were quantified using Pierce™ BCA (Thermo Fisher Scientific). ELISA assay for WNT5A quantification was performed using a commercially available ELISA kit (LS Bio) according to the manufacturer’s instructions.

***KD E4LEC and OE E4EC production.*** pGIPZ lentiviral shRNA against WNT5A gene (Dharmacon) and scrambled sequence control constructs encoding puromycin resistance and GFP were used. Lentiviral particles were generated in HEK293T cells using a second-generation packaging system. E4LECs were transfected with shRNA and scramble controls. Transduced cells were selected by 2.5 µg/ml Puromycin (Invitrogen) for 5-8 days and GFP-positive KD E4LECs were sorted by flow cytometry and transgene expression levels were quantified by RT-qPCR. pLX304 lentiviral vector (Dharmacon) that encodes a blasticidin resistance genes and WNT5A ORF expression from a cytomegalovirus (CMV) promoter was used to generate WNT5A overexpressed HUVECs. WNT5A lentivirus was produced in HEK293T cells and used to infect HUVECs. After infection, cells were selected by 2.5 µg/ml blasticidin (Invitrogen) for 5-8 days. WNT5A transduction was verified by RT-qPCR.

***Cell culture and colony forming assays.*** CD45^-^CD144^-^CD43^+^ CD235a^-^ cells from human fetal livers were sorted by FACS and cultured on E4orf1-transduced HUVECs (E4ECs), E4orf1-transduced liver ECs (E4LECs), E4orf1-transduced cardiac ECs (E4CECs), KD E4LEC and OE E4EC, as indicated, in serum-free StemSpan SFEM (StemCell Technologies) supplemented with Pen-Strep (Invitrogen), 100 µM monothioglycerol (MTG; Sigma-Aldrich), 50 μg/ml ascorbic acid, 50 ng/ml rhSCF (Peprotech), 20 ng/ml rhTPO (Peprotech), 20 ng/ml rhIL6 (Peprotech), 20 ng/ml rh IL3 (Peprotech), and 20 ng/ml rhFLT3L (Peprotech) for 10 days. Half the media was replaced with fresh media on day 3. Following 10 days of co-culture, non-adherent cells were resuspended by vigorous pipetting and passed through a 40 μm cell strainer (BD Falcon). The harvested cells were either analyzed by flow cytometry for phenotypic characterization or selected for CD34-positive cells via MACS for colony formation assays. The selected CD34+ populations were plated in complete methylcellulose medium containing human cytokines including SCF, GM-CSF, IL-3, G-CSF, EPO (Stem cell technologies), and supplemented with 20 ng/mL rhFLT3L (Peprotech) and 50ng/mL IL-6 (Peprotech). The plates were imaged, and colonies were analyzed at day 14 for total colony number, and each colony classified based upon morphologic criteria as granulocyte, erythrocyte, monocyte, megakaryocyte (CFU-GEMM), granulocyte/monocyte/macrophage (CFU-GM) or burst-forming erythroid (BFU-E). Colonies were stained with Giemsa using a Hematek II slide stainer following cytospin at 300 x g for 10 min and imaged on a Hamamatsu Nanozoomer Digital Pathology system.

***T cell differentiation.*** After co-culture with endothelial cells, non-adherent hematopoietic cells were resuspended by vigorous pipetting and passed through a 40 μm cell strainer (BD Falcon). The harvested cells were plated on bone marrow stromal cell line expressing notch ligand Delta-like 4 (OP9-Dll4) in α-MEM (Invitrogen) supplement with Pen-Strep (Invitrogen), 20% FBS (Hyclone), 100 µM monothioglycerol (MTG; Sigma-Aldrich), 50 μg/ml ascorbic acid, 50 ng/ml rhSCF (Peprotech), 20 ng/ml rhIL7 (Peprotech), and 20 ng/ml rhFLT3L (Peprotech) for 14-21 days. Floating hematopoietic cells were collected and stained with CD5 and CD7 for flow analysis to confirm the presence of T cells.

***Stromal free culture conditions.*** hESC differentiation was performed as described above in ‘Generation of hESC-derived hematopoietic progenitors’ up to day 5. On day 5, wells were harvested and co-cultured with OP9 cells in StemPro-34 supplemented with 100 µM MTG (Sigma-Aldrich), 50 μg/ml ascorbic acid, 50 ng/ml rhSCF (Peprotech), 20 ng/ml rhTPO (Peprotech), 20 ng/ml rh IL6 (Peprotech), 20 ng/ml rh IL3 (Peprotech), and 20 ng/ml rh FLT3L (Peprotech) for 4 days. Cells were then passaged to stromal free conditions on retronectin (5 μg/ml; Takara Bio) coated wells in serum-free StemSpan SFEM (StemCell Technologies) supplemented with 50 ng/ml rhSCF (Peprotech), 20 ng/ml rhTPO (Peprotech), 20 ng/ml rhIL6 (Peprotech), 20 ng/ml rh IL3 (Peprotech), and 20 ng/ml rhFLT3L (Peprotech) plus/minus 250 ng/ml rh Wnt5a (R&D Systems). Following 3 days of stromal free culture, cells were harvested for FACS analysis or CFU assay as described above.

***Statistical analysis.*** All data are presented as the mean ± error of the mean (SEM), and the results were analyzed using Prism Software (GraphPad, USA). A two-tailed Student’s t test was used to compare two groups. The one-way analysis of variance (ANOVA) with a Tukey’s post-hoc test was used to compare more than two groups.

**SUPPLEMENTARY FIGURES**


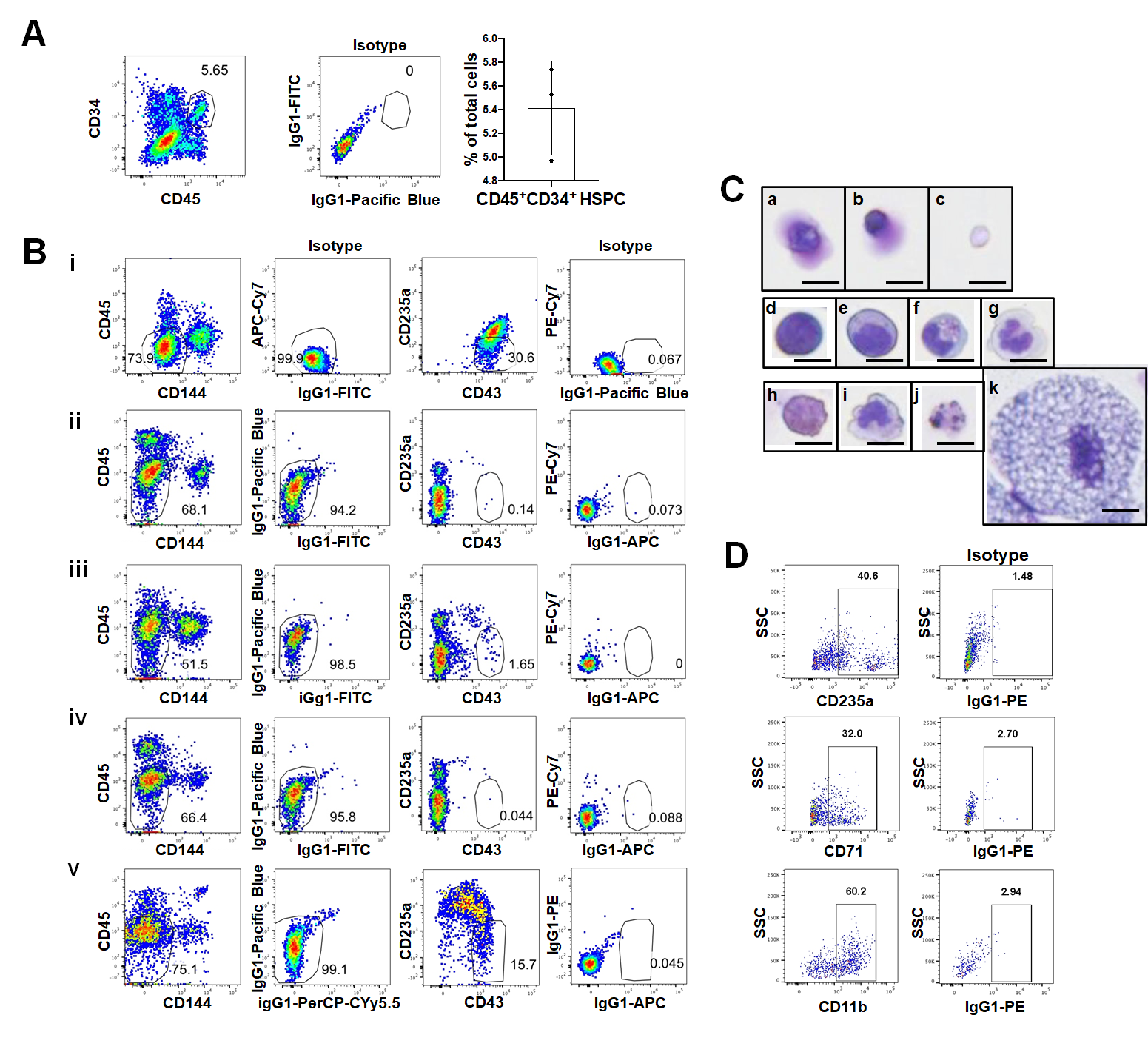


**Figure S1, A**. Phenotypic hematopoietic stem and progenitor cells (HSPCs, CD45^+^CD34^+^) were found to represent 5.4 ± 0.4 % (n = 3 donors, mean ± SEM) of total liver cells. **B**. Representative flow cytometry profiles of (i) hESC- HPCs with isotype controls, (ii-v) fresh total cell tissue suspension from fetal human kidney(ii), heart(iii), lung(iv) and liver(v) stained with markers for HPCs. **C**. Multiple hematopoietic cell types were identified including (a) polychromatic normoblasts, (b) orthochromic normoblasts, (c) polychromatic erythrocytes, (d) promyelocytes, (e) myelocytes, (f) metamyelocytes (g) polymorphonuclear neutrophils, (h) eosinophils, (i) monocytes, (j) basophils, and (k) macrophages (scale bar: 10 μm). **D.** Flow cytometry profiles of the colonies stained with erythroid and myeloid markers.


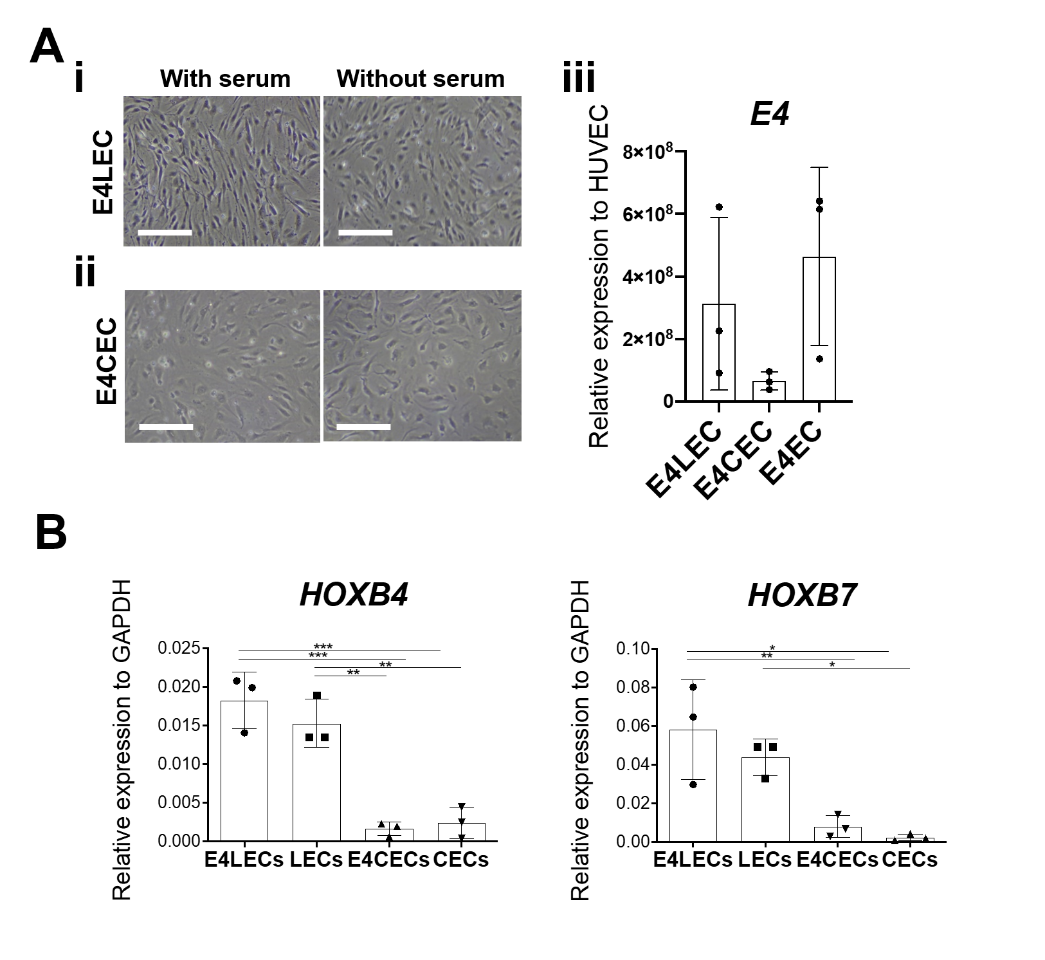


**Figure S2. A**. Bright field image of E4ORF1 transduced liver ECs (i) and heart ECs (ii) cultured with and without serum media. Scale bar: 200 μm. (iii) Ectopic expression of E4ORF1 in liver ECs (E4LECs), HUVECs (E4ECs), and cardiac ECs (E4CECs) compared with E4ECs was confirmed using a reverse-transcription quantitative PCR (RT-qPCR) (n=3 donors, mean ± SEM) **B**. Comparison of mRNA expression of selected genes for primary and E4ORF1 transfected heart and liver endothelial cells for liver endo-specific genes. (n=3 mean ± SEM * p≤0.05, **p≤0.01, ***p≤0.001)


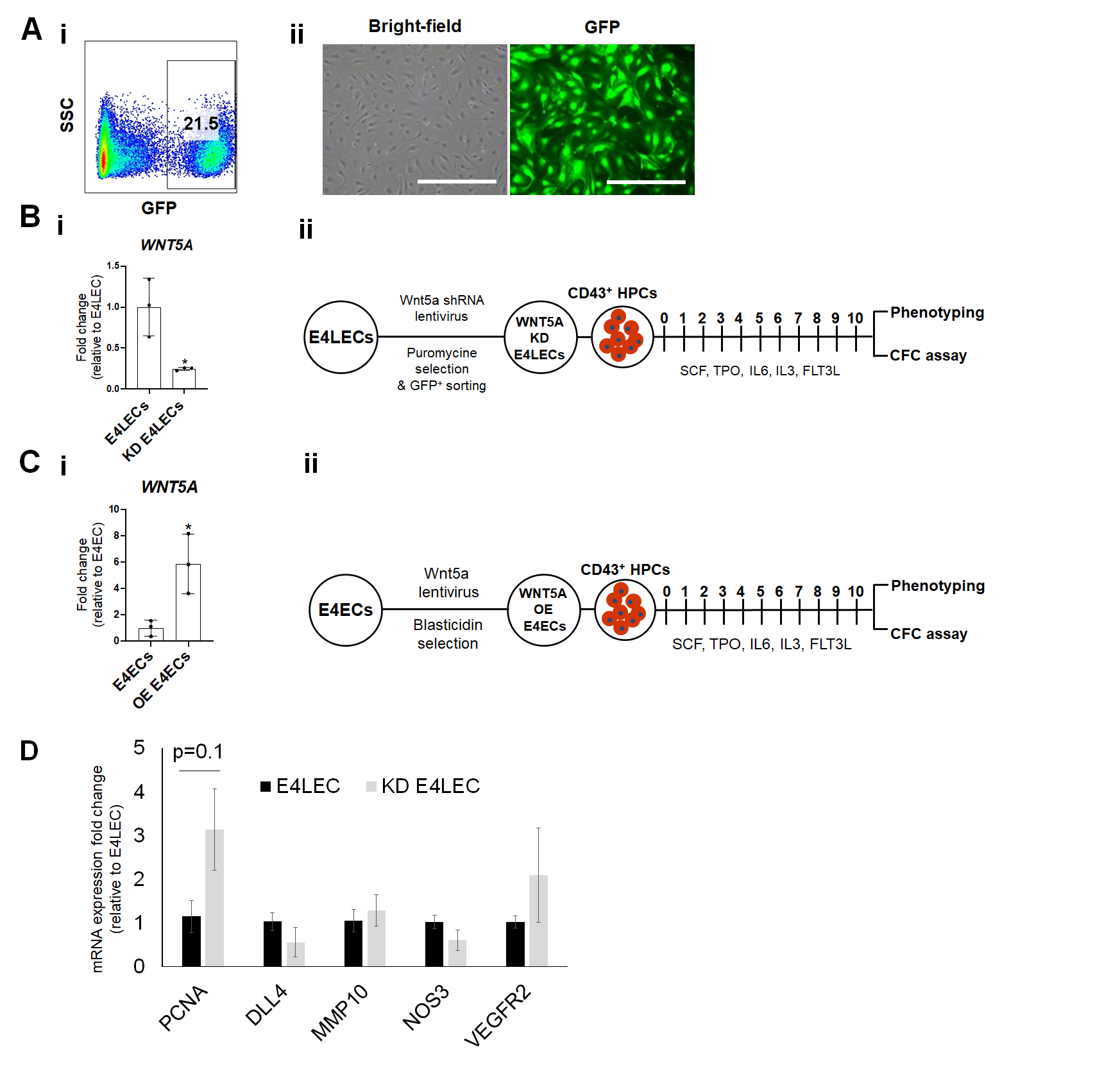


**Figure S3**. E4LECs were transduced with lentiviral vectors containing WNT5A (WNT5A-IRES-GFP) under the control of CMV promoter (i) FACS sorting scheme for GFP positive cells. (ii) Representative images of sorted GFP^+^ E4LECs. Scale bar: 0.5 mm. **B**. Knockdown of *WNT5A* on E4LECs. (i) RT-qPCR confirms *WNT5A* knockdown on E4LECs after transduction with pGIPZ lentiviral shRNA against *WNT5A* (n= 3 donors, mean ±SEM, * p≤0.05) (ii) Schematic diagram for preparation of KD E4LECs and coculture with liver HPCs for 10 days. **C**. Overexpression of *WNT5A* on E4ECs. (i)RT-qPCR confirms that *WNT5A* is overexpressed in E4ECs transduced with lentiviral vector that encodes blasticidin resistance genes and *WNT5A* gene. (n=3 mean ± SEM * p≤0.05) (ii) Schematic diagram of E4ECs cocultured with liver HPCs for 10 days. **D**. RT-qPCR showed that *WNT5A* knockdown on E4LECs led to non-significant changes in EC gene expression including *PCNA*, *DLL4*, *MMP10*, *NOS3* and *VEGFR2* (n= 3 donors, mean ±SEM)


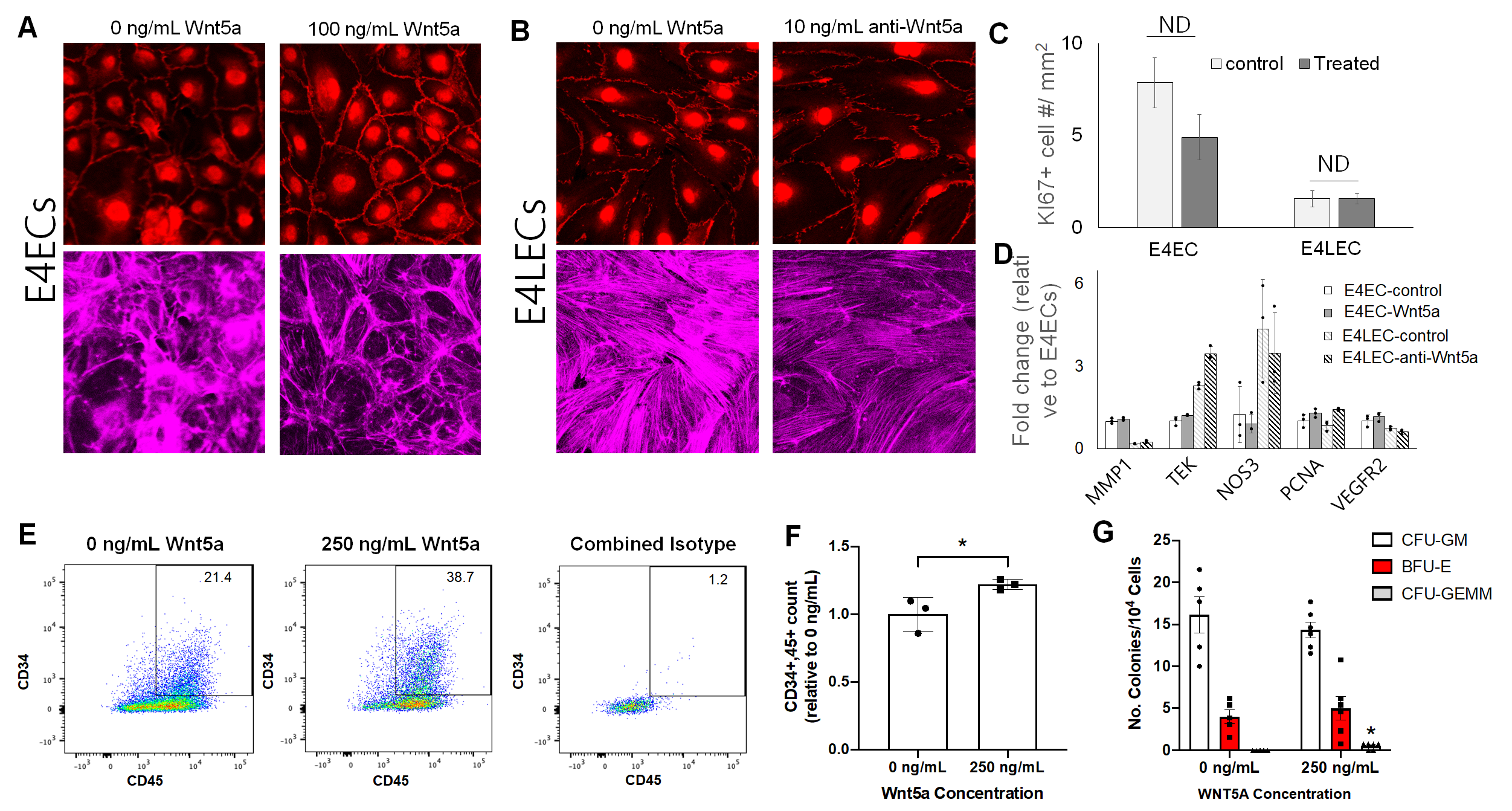


**Figure S4**. The role of soluble WNT5A on ECs and HPCs. **A-B**. Immunostaining of E4ECs culture at 0 or 100ng/mL WNT5A (A) and E4LECs at 0 or 10 ng/mL neutralization antibody for WNT5A (B) for 24 hours. Red: VE-Cad, and Magenta: F-actin. **C-D**. Quantification of Ki67+ cells (C) and expression of genes (D) for both ECs before and after respective treatments. **E-F**. Phenotyping (E-F) of stromal free culture of CD43+CD45-CD144- HSCs with or without WNT5A and CFU analysis (G) after 3 days of culture shows significant increase of CD34+ cells, and appearance of CFU-GEMM with soluble WNT5A ((n=3-6 mean ± SEM * p≤0.05).
